# Effectiveness of Diabetes Self-Management Education and Support on Glycemic Control and Diabetes-Related Outcomes in Africa: A Systematic Review and Meta-Analysis

**DOI:** 10.1016/j.focus.2025.100416

**Published:** 2025-08-20

**Authors:** Nuhu L. Adamu, Mohammed Merzah, Daisy Iminza, Kwang F. Tano, Taagbara Jolly Abaate, Parbati Thapa

**Affiliations:** 1 Department of Nursing Science, College of Medical Sciences, Modibbo Adama University, Yola, Girei, Nigeria; 2 Department of Public Health and Epidemiology, Faculty of General Medicine, University of Debrecen, Debrecen, Hungary; 3 Department of Community Health, Technical Institute of Karbala, Al-Furat Al-Awsat Technical University, Kufa, Iraq; 4 Department of Nursing Science, Kenyatta University Nairobi, Nairobi, Kenya; 5 Presbyterian Nursing & Midwifery Training College, Duayaw Nkwanta, Ghana; 6 School of Public Health, University of Port Harcourt, Port Harcourt, Nigeria; 7 Department of Community Medicine, University of Port Harcourt Teaching Hospital, Port Harcourt, Nigeria; 8 School of Health and Allied Sciences, Pokhara University, Lekhnath, Nepal

**Keywords:** DSMES, HbA1c, Type 2 diabetes, Africa, Glycemic control, Self-management

## Abstract

**Introduction:**

Diabetes prevalence in Africa continues to rise, presenting significant health and economic challenges. Effective management strategies, such as diabetes self-management education and support, are crucial for improving clinical outcomes. However, the impact of diabetes self-management education and support on glycemic control and related metabolic parameters in African populations remains unclear. This study evaluates the effectiveness of diabetes self-management education and support interventions compared with that of standard diabetes care in Africa, with a focus on glycemic control and secondary metabolic outcomes.

**Methods:**

A systematic review and meta-analysis were conducted in accordance with the PRISMA guidelines. Comprehensive searches were performed in PubMed, Embase, CINAHL, Scopus, Web of Science, and PsycINFO, supplemented with regional sources such as Africa Journal Online and ProQuest. RCTs published in English up to April 2024, involving adult participants with Type 2 diabetes in Africa who received diabetes self-management education and support interventions, were included. The Cochrane Risk of Bias Tool 1 was used to assess study quality. Heterogeneity was evaluated using I² statistics, and random-effects models were applied for pooled estimates.

**Results:**

Eighteen RCTs (2,599 participants) were included. diabetes self-management education and support interventions did not significantly improve glycemic control, with a pooled mean reduction in HbA1c of −0.25% (95% CI= −0.68, 0.18; *p*<0.0001). Fasting blood glucose outcomes, assessed in 2 studies, showed no significant difference (mean difference= −0.13, 95% CI= −0.37, 0.12). Secondary outcomes (BMI, blood pressure, lipids) showed no significant changes. Sensitivity analysis excluding 1 study showed a larger effect size (−0.42%, 95% CI= −0.76, −0.07). Substantial heterogeneity (I²=93.8%) was observed.

**Conclusions:**

Diabetes self-management education and support reveals a nonsignificant impact on HbA1c reduction in African adults with Type 2 diabetes and emphasizes the need for more standardized approaches and broader outcome measures. Future studies should aim to refine diabetes self-management education and support models, enhance patient engagement, and evaluate a wider range of health outcomes to realize the full potential of diabetes self-management education and support in resource-limited settings.

## Highlights

• Diabetes self-management education and support did not significantly reduce HbA1c in African patients with diabetes.
• Meta-analysis included 18 RCTs from diverse African countries.
• High heterogeneity was observed in glycemic outcome measures.
• No significant effects on BMI, blood pressure, or lipid profiles were observed.
• Standardized diabetes self-management education and support models are needed in low-resource settings.

## INTRODUCTION

Diabetes is a significant public health concern worldwide, with over half a billion adults currently affected.^1^ More than three quarters of people living with diabetes reside in low- and middle-income countries, where healthcare systems often face challenges in addressing the complexities of chronic disease management.^1^ The African region is no exception, witnessing a significant rise in diabetes prevalence. Approximately 1 in 22 adults in the area were diagnosed with diabetes in 2021, with many more undiagnosed.^2^ This escalating burden underscores the need for comprehensive strategies to combat the disease and its associated complications.

Effective diabetes management demands a multidisciplinary approach, involving healthcare providers; family members; and, most importantly, the individuals themselves.^3^ Poorly managed diabetes can lead to severe complications, including cardiovascular diseases, kidney failure, and neuropathy, which significantly reduce quality of life and increase healthcare costs.^4^ Diabetes self-management education and support (DSMES) has emerged as a cornerstone of diabetes care. The American Diabetes Association defines DSMES as an evidence-based intervention that equips individuals with the knowledge, skills, and confidence needed to manage their condition effectively.^5^ DSMES promotes informed decision making, problem solving, and sustainable self-care behaviors by fostering collaboration between patients and healthcare teams. Studies have consistently demonstrated that individuals participating in DSMES programs achieve better glycemic control, improved quality of life, and reduced healthcare costs compared with those who receive standard care alone.^6^

Despite its proven benefits in high-income settings, the implementation of DSMES in Africa faces unique challenges. The continent’s diverse cultural, linguistic, and socioeconomic contexts complicate the delivery of standardized education programs.^7^ Limited healthcare resources, a shortage of trained professionals, and low levels of health literacy further hinder the adoption of DSMES.^7^ However, the potential of DSMES to address these challenges is immense, particularly when interventions are tailored to local needs. For instance, community-based and peer-led models have shown promise in overcoming barriers to accessibility and affordability.^8^

Existing evidence on DSMES in Africa is limited but growing. Preliminary studies suggest that DSMES can improve diabetes-related outcomes, such as HbA1c levels and self-care behaviors, although results are often inconsistent owing to variations in intervention design and delivery.^9^ This systematic review and meta-analysis aims to synthesize available data on the effectiveness of DSMES in African settings to evaluate the effectiveness of DSMES on glycemic control and other diabetes-related clinical outcomes in patients with Type 2 diabetes and recommend actionable strategies to improve diabetes care across the region.

## METHODS

A systematic review and meta-analysis following the PRISMA chart was conducted to evaluate the effectiveness of DSMES on clinical outcomes among patients with Type 2 diabetes in Africa. The review included only RCTs published in the English language. The review was registered with PROSPERO (Registration Number CRD42023460051).

The authors conducted a comprehensive literature search up to April 2024 to identify relevant studies evaluating the effectiveness of DSMES in Africa. The search was conducted across major electronic databases, including PubMed, Embase, CINAHL, Scopus, Web of Science, and PsycINFO, with the inclusion of regional sources such as *Africa Journal Online* and ProQuest for gray literature.

The search strategy employed a combination of Medical Subject Headings terms and free-text keywords to capture all variations of DSMES interventions. Search terms included combinations of *diabetes self-management education, DSMES, self-care education*, and *self-management support* alongside terms such as *Type 2 diabetes, T2DM, Africa, Sub-Saharan Africa*, and specific country names. Boolean operators were utilized to enhance the search scope. Manual searches of reference lists from relevant studies and citation tracking were also conducted to identify additional studies.

Studies were eligible for inclusion if they met the following criteria: (1) conducted in African settings, (2) focused on adult populations (aged ≥18 years) with Type 2 diabetes, (3) included structured DSMES interventions, (4) reported at least 1 clinical outcome such as HbA1c, and (5) published in the English language. Studies without a comparator group or lacking baseline and follow-up data were excluded.

To ensure methodologic rigor and minimize bias, the predefined inclusion criteria restricted the review to RCTs. Although some relevant nonrandomized studies were initially identified, they were excluded during the screening phase. The authors recognize that this approach may have led to the omission of valuable evidence from well-conducted nonrandomized studies, a limitation that is addressed in the discussion section.

To minimize selection bias, 2 independent reviewers screened the titles and abstracts of the identified studies. The full texts of potentially eligible studies were then assessed against the inclusion criteria, with discrepancies resolved through discussion or consultation with a third reviewer. Covidence software was employed to streamline the screening and data extraction processes, reducing human error and ensuring consistency. All extracted data were cross-verified by 2 reviewers, focusing on study characteristics, intervention components, and outcomes.

All identified citations were loaded into the EndNote referencing software, and duplicates were checked and autoremoved. The references were imported into Covidence software. The titles and abstracts were screened by 2 independent reviewers, and full texts were retrieved automatically by the Covidence software, with only a few imported manually. In the same manner, the eligible studies included in the full text were critically assessed by 2 independent reviewers. Reasons for exclusion of full-text studies that did not meet the inclusion criteria were recorded and reported. In both cases, discrepancies were resolved by consensus and a third reviewer.

All statistical analyses were conducted according to a prespecified plan developed prior to data extraction. Meta-analyses were conducted using the random-effects model to account for anticipated heterogeneity. This model was chosen a priori, recognizing the clinical and methodologic diversity across the included trials. The authors assessed statistical heterogeneity among studies using the *I*² statistic, which quantifies the percentage of total variation across studies due to heterogeneity rather than chance. An I² value of 0%–25% was considered low heterogeneity, that of 26%–50% was considered moderate heterogeneity, and that >50% was considered substantial heterogeneity.

Sensitivity analysis was preplanned to test the robustness of the primary outcome by excluding studies with a high risk of bias or statistical influence. Although no specific quantitative thresholds were predefined for exclusion, the authors assessed studies posthoc using visual inspection of forest plots and heterogeneity diagnostics. In particular, studies exhibiting extreme effect sizes and a noticeably high influence on pooled estimates (based on their CI width and visual leverage on the summary effect) were considered for exclusion. This approach was applied cautiously to explore the impact of influential studies on the overall findings. A funnel plot was used to assess potential publication bias; however, no formal tests for asymmetry were conducted owing to the limited number of included studies per outcome. Analyses were performed using R software 4.1.0. All statistical tests were 2 sided, and a *p*<0.05 was considered statistically significant.

Data were extracted by 2 independent members of the review team using the Covidence Data Extraction Tool 1 for intervention studies. The data extraction was based on the review question/objectives and the inclusion criteria. This included the study participants, settings, interventions, comparators, outcome measures, results, and other relevant data as captured in the data extraction tool. Quality assessment of included studies was assessed using the Cochrane Risk of Bias Tool 1 (Appendix Table 1, available online), focusing on the following 7 criteria: sequence generation, allocation concealment, blinding of participants and personnel, blinding of outcome assessors, incomplete outcome data, selective outcome reporting, and other sources of bias. Each criterion was judged as low, high, or unclear. Each study’s methodologic rigor was critically appraised, and publication bias was assessed through funnel plot symmetry (Appendix Figure 1, available online).

The characteristics of the included studies captured in Table 1^9^, ^10^, ^11^, ^12^, ^13^, ^14^, ^15^ present the following highlights:

1. Geographic diversity: Studies were conducted across multiple countries, including Kenya, Tanzania, South Africa, Nigeria, Ethiopia, Rwanda, Egypt, Ghana, and Mali.
2. Study designs: All the studies utilized RCT designs, ensuring high internal validity for the reported outcomes. Sample sizes ranged from 77 to 275 participants, capturing a broad spectrum of participants with diverse demographic and clinical characteristics. The interventions included DSMES delivered by nurses, family integration, peer-led education, structured nutrition programs, intensive patient education, pharmacist-led interventions, and technology such as short message service (SMS)–based reminders.
3. Inclusion criteria: Most studies included adult participants diagnosed with Type 2 diabetes, with HbA1c thresholds above 7% or 8%. Some studies required participants to demonstrate the ability to engage with specific intervention components, such as using SMS services or attending educational sessions.

**Table 1:** Characteristics of the Included Studies

Author/date	Country	Study aim	Total sample size (intervention/control allocated)	Intervention	Inclusion criteria	Comparison groups	Duration of the interventions	Follow-up periods
Gathu et al.,^14^ 2018	Kenya, United Republic of Tanzania	To assess the effects of DSME in comparison with those of usual diabetes care by family physicians	140 Intervention: 70 (8.9±1.89) Control: 70 (9.3±1.75)	Controlled DSME	Recruited and screened patients from the FMC diabetes registry who had suboptimally controlled T2D, defined as HbA1c >8%, and aged 18–65 years	Standard diabetes care	One-hour education session every 6 weeks for 4 months	6 months
Muchiri et al.,^12^ 2016	South Africa	To evaluate the effect of a participant-customized NEP on HbA1c.	82 Intervention: 41 (9.8±0.3) Control: 41 (10.4±0.3)	Received education materials and participated in the NEP	At least 1 year of living with diabetes; regular attendance at the CHCs; blood sugar levels >10 mmol/L on 2 occasions in the previous 6 months and consequent HbA1c levels >8% after blood analysis; nonpregnant and not on insulin therapy	The control group received education materials and continued with the usual medical care	Two-hour education session every 8 weeks for 4 months	12 months
Essien,^16^ 2017	Nigeria, the United Kingdom, and Northern Ireland	Intensive patient education improves glycemic control in diabetes compared with conventional education	118 Intervention: 59 (10.9±1.7) Control: 59 (10.5±1.5)	Intensive patient education program	Eligible patients (aged ≥18 years, HbA1c >8.5%, and physically able to participate) were randomly allocated by permuted block randomization to participate for 6 months in either an intensive or conventional education group.	Conventional education (there were no mobile phone reminder messages).	Two-hour education fortnightly over 6 months (12 structured teaching sessions)	6 months
Muchiri,^17^ 2021	South Africa	To investigate the effectiveness of the adapted structured nutrition education program on clinical status, dietary behaviors, and behavior mediators in adults with poorly controlled diabetes	77 Intervention: 39 (9.91±0.2) Control: 38 (9.92±0.2)	Diabetes education materials and NEP	Patients with diabetes aged 40–70years, HbA1c ≥8%, living with diabetes for at least 1 year, ability to understand English, not pregnant	Diabetes education materials only.	Seven monthly group education sessions, bimonthly group follow-up, and 1 individual counseling session. All interventions were completed within 1 year.	12 months
Ojewale,^18^ 2022	Nigeria	To determine the effects of family-integrated diabetes education on the diabetes knowledge of patients and family members as well as its impact on HbA1c	170 Intervention: 88 (7.8±2.1) Control: 82 (7.5±1.8)	Family-integrated education program and SMS	PLWD and family members aged ≥18 years and without cognitive impairment were placed, as clusters, into either a CG or an IG	Standard care	Weekly education program for four weeks.	6 months
Diriba et al.,^15^ 2023	Ethiopia	To examine the preliminary effects of a culturally tailored, family-supported, community-based DSMES program for T2D on HbA1c, blood pressure, BMI, and lipid profiles	80 Intervention: 40 (62±7.8) Control: 40 (72±8.7)	Culturally tailored DSMES	People with T2D were included if they (1) were aged ≥18 years, (2) lived in 2 selected Kebeles in Nekemte, (3) were able to nominate primary family caregivers who could support them in diabetes management, and (4) were taking insulin and/or oral hypoglycemic agents.	Usual care	Six 2-hour education programs	2 months
Ng'ang'a,^19^ 2022	Rwanda	To assess the feasibility and effectiveness of implementing SMBG among patients with insulin-dependent T2D receiving care in 3 rural district hospitals in Rwanda.	80 Intervention: 42 (7.05±1.61) Control: 38 (7.99±2.38)	Diabetes education with an SMBG kit to implement SMBG at home	Adults aged at least 18 years diagnosed with insulin-dependent T2D and receiving an insulin regimen at the time of study at 1 of the 3 district hospitals listed earlier were eligible. Eligible participants must have had the most recent HbA1C recording at 7% or greater.	Usual care	Weekly education for 3 months	6 months
Abaza,^20^ 2017	Egypt	To examine the feasibility of SMS education among patients with diabetes in Egypt and assess the impact of educational text messages on glycemic control and DSM	90 Intervention: 45 (9.78±2.53) Control: 45 (9.53±2.78)	Diabetes care instruction booklet, educational SMS messages	Patients were included if they had diabetes, owned a mobile phone, and could read SMS messages or lived with someone who could read for them.	Diabetes care instruction booklet only	Daily educational SMS messages for 12 weeks	3 months
Eshete et al.,^10^ 2023	Ethiopia	To evaluate nutritional promotion interventions for dietary adherence and lessons learned to improve self-management.	216 Intervention: 108 (172.13±54.81) Control: 108 (186.38±54.95)	Physical activity and nutrition promotion program	Patients aged 20–70 years, with no complications, who stayed for at least 6 months and had no intention of leaving	Usual care	Weekly education for 2 months	6 months
Kiarie,^21^ 2024	Kenya	To assess the comparative effectiveness of improved primary caregiver social support capacity on self-management practices of clients with T2D in Machakos County, Kenya	275 Intervention: 137 (8.93±3.55) Control: 138 (10.55±5.72)	Implementing a diabetes care plan	Clients diagnosed with T2D and enrolled in government-owned public health facility diabetes care and treatment programs in the Masinga and Matungulu subcounties, aged between 18 and 65 years, who could read and write, and who lived with or near a person aged >18 years who could serve as a primary caregiver	Usual care	Monthly self-management education for 6 months	6 months
Lamptey et al.,^11^ 2023	Accra, Ghana	Change in glycemic control with structured DSM education in urban low-resource settings	206 Intervention: 103 (7.3± −1.5) Control: 103 (7.3± −1.5)	Structured DSME	Eligibility criteria included age ≥18 years, ability to participate in activities in a group setting, and being known to have T2DM and not known to have chronic kidney or sickle cell disease	Usual care only	Six hours of intensive group education	3 months
Debussche et al.,^9^ 2018	Mali	To evaluate the effectiveness of peer-led self-management education in improving glycemic control in patients with T2D in a low-income country	151 Intervention: 76 (10.6±1.8) Control: 75 (10.8±1.9)	Peer-led DSME	Patients included in the study were aged between 30 and 80 years, underwent regular follow-ups and monitoring in Bamako consultation units for poorly controlled T2D (HbA1c>8%), and agreed to have their clinical and biological measurements taken until completion of the protocol.	Standard care	1–2 hours peer education course delivered monthly for 9 months	12 months
David,^22^ 2021	Nigeria	To evaluate the impact of pharmacist-led- led care on glycemic control in patients with uncontrolled T2D	108 Intervention: 54 (7.3±none) Control: 54 (8.0±none)	Pharmacist-led diabetes education intervention	(1) Clinically diagnosed patients with T2DM with greater than or equal to 7% HbA1c, (2) patients with at least 6 months of regular clinic attendance before recruitment, and (3) patients who were aged ≥18 years	Usual care	30–45 minutes of initial education and at 3 months. Reinforce phone calls and SMS between	6 months
Hailu,^23^ 2019	Ethiopia, Norway	To develop and test the effectiveness of a multifaceted, nurse-led DSME program for improving diabetes knowledge, self-care activities, and self-efficacy in an Ethiopian setting	220 Intervention: 116 (11.33±0.25) Control: 104 (10.61±0.27)	Nurse-led DSME	Patients with T2DM aged ≥30 years at the time of diagnosis and who had used or were presently taking oral hypoglycemic agents or insulin were eligible for inclusion in the study.	Routine diabetes care	Six interactive DSME sessions once a month for 6 months	9 months
Muchiri et al.,^12^ 2016	South Africa	To evaluate the effect of a nutrition education program on diabetes knowledge and attitudes of adults with T2DM	82 Intervention: 41 (9.8±0.03) Control: 41 (10.4±0.03)	Received education materials (pamphlet and wall/fridge poster) and participated in the NEP	At least 1 year of living with diabetes, regular attendance at the CHCs, blood sugar levels of 10 mmol/L on 2 occasions in the previous 6 months and consequent HbA1c levels of 8% after blood analysis, nonpregnant and not on insulin therapy	Education materials only and usual care	2 hours of weekly nutrition education for 8 weeks	12 months
Hailu,^24^ 2021	Ethiopia	To evaluate the effect of a locally contextualized, nurse-led DSME program on psychosocial health and quality of life among people with T2D in Ethiopia	220 Intervention: 116 (27.0±9) Control: 104	Nurse-led DSME	Patients with T2DM aged ≥30 years at the time of diagnosis and those who had used or were presently taking oral hypoglycemic agents or insulin were eligible for inclusion in the study	Standard care	Six DSME sessions once a month for 6 months	3 months
Tamiru,^25^ 2023	Ethiopia	To assess the effect of DSME on self-care knowledge and behavior among adult patients with diabetes	360 Intervention: 180 (11.23±0.31) Control: 180 (8.21±0.29)	DSME	Registered patients aged ≥18 years with T2DM attending diabetes follow-up clinics	Routine diabetes care	One hour of monthly education for 6 months	6 months
Githinji et al.,^13^ 2022	Kenya, U.S.	To improve diabetes knowledge, health beliefs, dietary intake, physical activity, and weight status among Kenyan adults	226 Intervention: 116 (2.87±0.43) Control: 110 (1.4±0.69)	Diabetes education	Adults aged ≥18 years with T2D, living within periurban communities in Embakasi constituency	Standard care	3 hours daily education for 5 days, followed by phone SMS for 4 weeks	3 months

CG, control group; CHC, Comprehensive Health Centre; DSM, diabetes self-management; DSMES, diabetes self-management education and support; FMC, Federal Medical Center; IG, intervention group; NEP, nutrition education program; PLWD, people living with diabetes; SMBG, self-monitoring of blood glucose; SMS, short message service; T2D, Type 2 diabetes; T2DM, Type 2 diabetes mellitus.

## RESULTS

**Figure 1:**
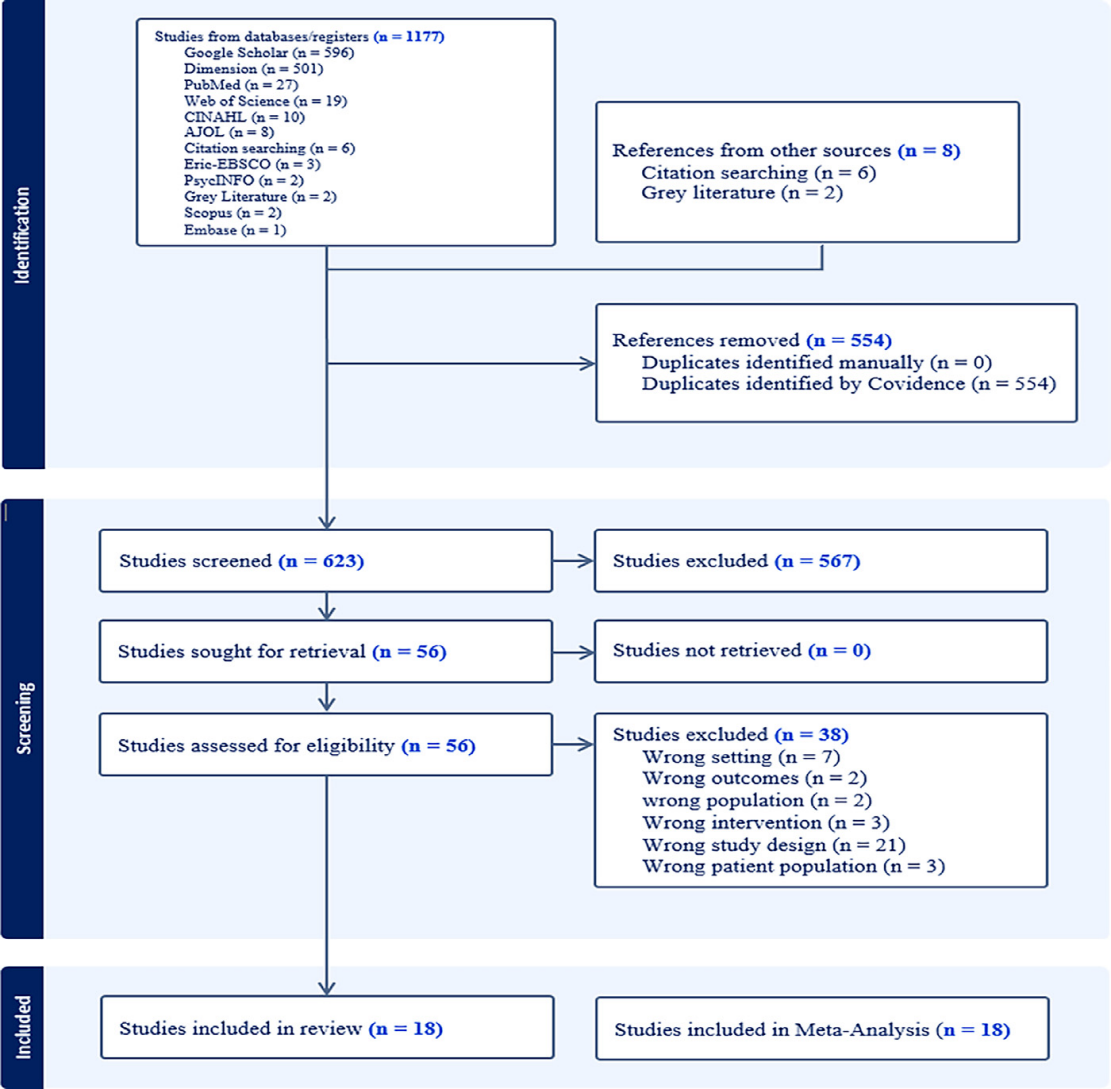
PRISMA flow chart.

At the identification level, a total of 1,177 studies were imported for screening. In the screening phase, there were 623 studies reviewed, and 567 studies were found to be irrelevant and excluded from further consideration. A total of 56 studies were assessed for eligibility in the full text, and 38 studies were excluded for various reasons, which included wrong study design, wrong setting, wrong patient population, wrong intervention, or wrong outcome (Appendix Table 2**,** available online). Finally, 18 studies (Table 1), encompassing 2599 participants, were included in the final analysis (Figure 1).

**Figure 2:**
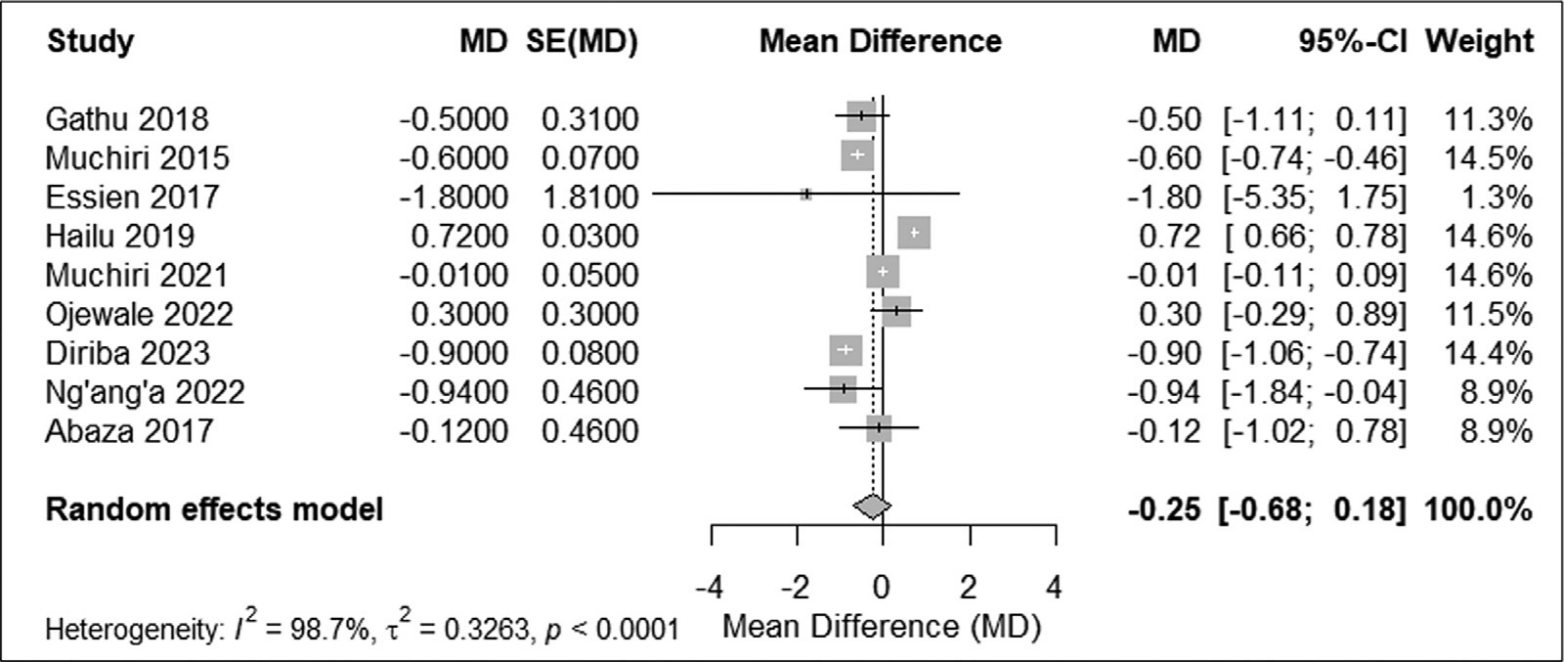
Meta-analysis of the effect of DSMES interventions on HbA1c levels in patients with diabetes. DSMES, diabetes self-management education and support.

Of 18 studies, 11 reported glycemic outcomes (9 studies on HbA1c and 2 studies fasting blood sugar). The pooled effect size for HbA1c, on the basis of a random-effects model across 9 studies, was −0.25 (95% CI= −0.68, 0.18), indicating no statistically significant improvement in glycemic control after DSMES interventions. Although the *p*-value for heterogeneity was <0.0001, reflecting substantial between-study variability, the primary analysis did not demonstrate a significant reduction in HbA1c (Figure 2).

**Figure 3:**
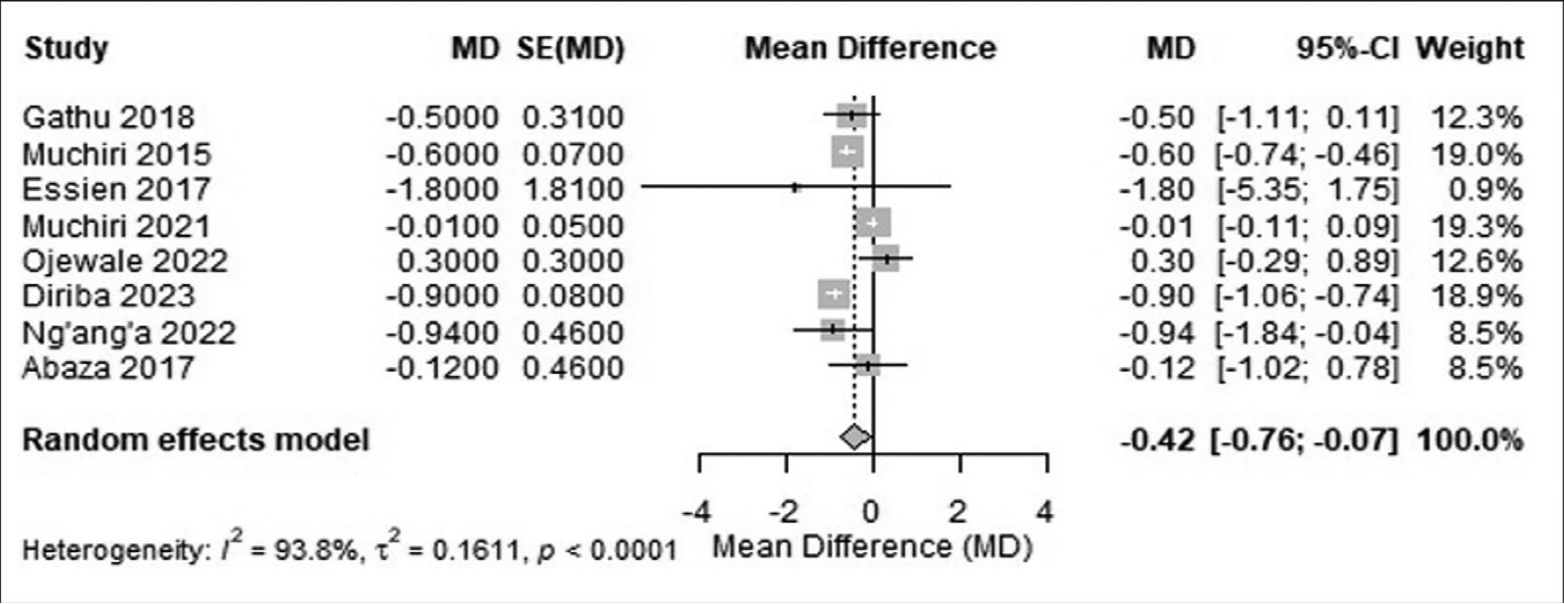
Sensitivity analysis of the effect of DSMES interventions on HbA1c levels. DSMES, diabetes self-management education and support.

In the sensitivity analysis, after excluding the study by Hailu (2019), the meta-analysis demonstrated a pooled mean difference (MD) of −0.42 (95% CI= −0.76, −0.07), suggesting a statistically significant reduction in HbA1c levels among participants in the intervention group compared with that among the control group despite heterogeneity (I²=93.8%, *I*^2^=0.1611, *p*<0.0001) (Figure 3). In the sensitivity analysis, the authors excluded the study by Hailu (2019) owing to its high influence and statistical leverage, identified through visual inspection of the forest plot and heterogeneity diagnostic.

**Figure 4:**
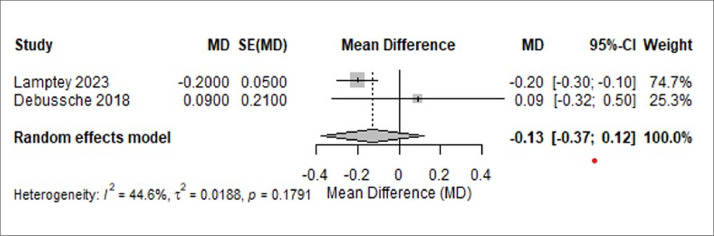
Meta-analysis of the effect of DSMES interventions on fasting blood glucose. DSMES, diabetes self-management education and support.

Further analysis of 2 studies reporting fasting blood glucose values compared patients with diabetes who self-managed with those who did not. The random effects model yielded an overall MD of −0.13 (95% CI= −0.37, 0.12), which was not statistically significant. Heterogeneity among the studies was moderate (I²=44.6%, *p*=0.1791), indicating variability in the study outcomes (Figure 4).

**Figure 5:**
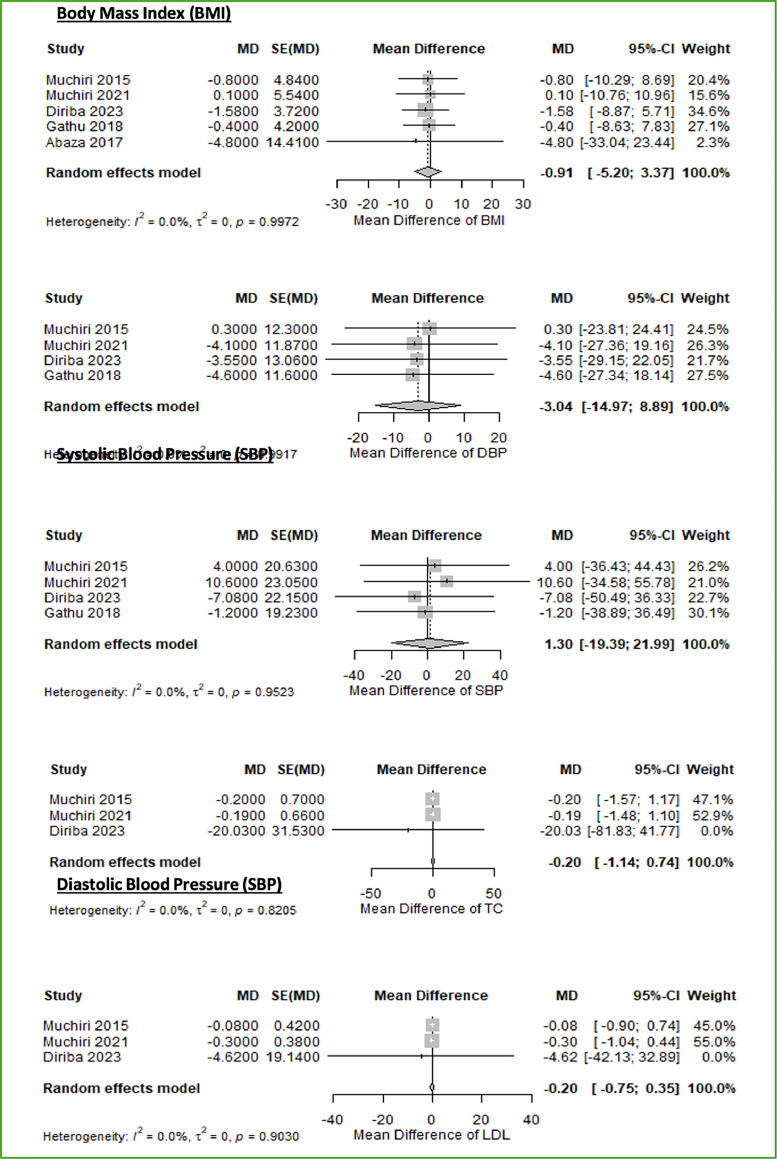
Meta-analysis of the effect of DSMES interventions on other outcomes in patients with diabetes. DSMES, diabetes self-management education and support.

The meta-analysis also examined the effects of self-management interventions on additional metabolic and cardiovascular parameters, including BMI, blood pressure, total cholesterol, and low-density lipoprotein levels. The pooled MD from 3 studies for BMI was −0.91 (95% CI= −5.20, 3.37), indicating no significant reduction. Similarly, no significant effects were observed from 3 studies for systolic blood pressure (MD= −3.04, 95% CI= −14.97, 8.89) and 2 studies for diastolic blood pressure (MD=1.30, 95% CI= −19.39, 21.99). Regarding lipid profiles, total cholesterol showed a pooled MD of −0.20 (95% CI= −1.14, 0.74) from 2 studies, and another 2 studies showed a pooled MD of 0.20 (95% CI= −0.75, 0.35) for low-density lipoprotein levels, suggesting no significant changes. The heterogeneity (I²) across these outcomes remained low, indicating consistency across studies (Figure 5).

Visual inspection of the funnel plot (Appendix Figure 1, available online) indicated a relatively symmetrical distribution of effect sizes, suggesting a low likelihood of publication bias. However, owing to the limited number of studies, particularly for secondary outcomes, this should be interpreted with caution.

## DISCUSSION

The findings of this systematic review and meta-analysis highlight the potential of DSMES interventions to improve diabetes-related outcomes in African settings. However, the present meta-analysis evaluated the impact of DSMES interventions on glycemic control, with pooled data from 9 studies indicating a nonsignificant reduction in HbA1c. Despite growing interest in DSMES as a holistic approach to diabetes care, the findings suggest limited efficacy in uniformly improving glycemic outcomes. This result contrasts with global studies, which have established DSMES as a cornerstone of effective diabetes care.^5^^,^^14^^,^^26^ Similarly, the meta-analysis of the 2 studies that reported fasting blood glucose was not statistically significant. Given that only 2 studies reported on fasting blood glucose, the pooled analysis is limited in its interpretability.

Several factors may account for this result. First, the substantial heterogeneity across studies highlights methodologic variability, including differences in intervention design, duration, delivery mode, and patient demographics. Such inconsistencies could attenuate observable effects and mask the benefits of DSMES in specific contexts. Second, variations in adherence and participant engagement may further dilute treatment effects because DSMES relies heavily on sustained behavioral changes. It is also possible that the metrics used (primarily HbA1c) may not fully capture the breadth of DSMES’ impact, which can extend to self-efficacy, psychological well-being, and healthcare utilization. Future research might consider multidimensional outcome assessments and explore which DSMES components yield the most consistent benefits.

The substantial heterogeneity in study outcomes suggests variability in the implementation, content, and delivery methods of DSMES programs.^27^ This highlights the need for standardized, culturally adapted interventions that can be widely adopted in diverse African settings. For instance, interventions incorporating community-based, family supported, or peer-led models have shown promise in addressing unique cultural and socioeconomic barriers in Africa.^7^^,^^12^ These models leverage local resources and social networks to enhance patient engagement and adherence, making DSMES more accessible and sustainable in low-resource settings.

In the same vein, the study shows limited significant changes in secondary outcomes, such as BMI, blood pressure, and lipid profiles, suggesting that DSMES interventions may require additional components, such as dietary modifications and physical activity promotion, to yield broader metabolic benefits.^10^^,^^15^

The inconsistent effects observed in fasting blood glucose measurements further suggest that DSMES efficacy may depend on participant adherence, duration of intervention, and the extent of healthcare support provided.^28^ The high level of heterogeneity in glycemic outcomes (I²=93.8%) may be attributed to differences in study design, sample size, intervention duration, and participant characteristics.^29^ These variations emphasize the need for further research to identify key components of effective DSMES programs and optimize their delivery to enhance consistency and impact.

Several barriers to DSMES implementation in Africa persist. Limited access to trained diabetes educators, inadequate funding, and low health literacy among patients are significant obstacles.^7^^,^^11^ Innovative solutions, such as the use of mobile health technologies, could help bridge these gaps. For example, SMS-based reminders and telehealth consultations have been shown to improve patient adherence and reduce costs, making them viable options for scaling up DSMES interventions.^30^

This review included only RCTs, which may have excluded relevant evidence from nonrandomized studies that could offer additional insight into real-world effectiveness. Furthermore, the restriction to studies published in English may have introduced language bias, potentially omitting relevant studies from Francophone, Lusophone, or Arabophone African countries. The lack of long-term follow-up data in most studies limits the ability to assess the sustained impact of DSMES on diabetes outcomes. Future reviews could consider multilingual search strategies or collaborations with experts fluent in Arabic, French, and Portuguese to enhance coverage of studies on the African continent.

### Limitations

The high heterogeneity observed in HbA1c outcomes (I²=93.8%) highlights substantial variability across studies, likely due to differences in intervention formats, duration, cultural tailoring, educator training, and baseline glycemic status. The authors did not conduct subgroup analyses or meta-regression owing to the limited number of trials per covariate, which limits the ability to explore sources of heterogeneity. The analysis of fasting blood glucose included only 2 studies, limiting statistical power and precision. The moderate heterogeneity (I²=44.6%) also complicates interpretation. There is a need for standardized reporting in future primary studies to facilitate accurate analysis. In addition, most studies lacked long-term follow-up, making it difficult to assess the durability of DSMES effects over time. Future studies should incorporate follow-up periods of 12 months or more. Although the authors used a funnel plot to explore publication bias, no formal asymmetry tests were conducted because the small number of studies per outcome limits their statistical power. In addition, the meta-analysis for fasting blood glucose was based on only 2 studies, reducing the robustness of the conclusions for that outcome. The authors were also unable to conduct subgroup analyses or meta-regression to explore potential sources of heterogeneity owing to the lack of consistent subgroup-level data across studies. Furthermore, most included studies lacked long-term follow-up, limiting the ability to assess the durability of intervention effects. Finally, cost-effectiveness analyses are needed to inform resource allocation and policy decisions, particularly in low-resource settings.^8^^,^^13^

## CONCLUSIONS

Although DSMES remains a promising strategy for empowering individuals with diabetes, the current evidence in this study does not support a statistically significant improvement in glycemic control on the basis of pooled HbA1c data. The high heterogeneity underscores the need for more standardized, context-sensitive interventions and better-defined outcome measures. However, successful implementation requires the incorporation of DSMES as a standard component of primary healthcare services, addressing systemic barriers, including training for educators, resource allocation, and cultural adaptations. Policymakers and healthcare providers should invest in scalable DSMES programs to mitigate the growing diabetes burden across the region.

Therefore, future studies should aim to refine DSMES models, enhance patient engagement, and evaluate a broader range of health outcomes to realize their full potential in diabetes management. Researchers should also focus on evaluating cost-effective delivery methods; addressing the long-term impact of DSMES on glycemic control and quality of life; and developing standardized metrics for measuring secondary outcomes such as BMI, blood pressure, and lipid profiles.

## Appendix Supplementary materials

Image, application 1

## Footnotes

Supplementary material associated with this article can be found in the online version at doi:10.1016/j.focus.2025.100416.

## References

[1] International Diabetes Federation (2021). IDF Diabetes Atlas.

[2] World Health Organization, Regional Office for Africa (2023).

[3] Davis J., Fischl A.H., Beck J. (2022). 2022 National standards for diabetes self-management education and support. Diabetes Care.

[4] ElSayed N.A., Aleppo G., Aroda V.R. (2023). Introduction and methodology: standards of care in Diabetes-2023. Diabetes Care.

[5] Powers M.A., Bardsley J.K., Cypress M. (2020). Diabetes self-management education and support in adults with type 2 diabetes: a consensus report of the American Diabetes Association, the association of diabetes care & education specialists, the academy of nutrition and dietetics, the American Academy of Family Physicians, the American academy of PAs, the American Association of nurse practitioners, and the American Pharmacists Association. Diabetes Care.

[6] Association of Diabetes Care and Education Specialists (ADCES) (2023).

[7] Kumah E., Otchere G., Ankomah S.E. (2021). Diabetes self-management education interventions in the WHO African Region: a scoping review. PLoS One.

[8] American Diabetes Association Professional Practice Committee (2024). 1. Improving care and promoting health in populations: standards of care in diabetes-2024. Diabetes Care.

[9] Debussche X., Besançon S., Balcou-Debussche M. (2018). Structured peer-led diabetes self-management and support in a low-income country: the ST2EP randomised controlled trial in Mali. PLoS One.

[10] Eshete T., Lambebo A., Mohammed S., Shewasinad S., Assefa Y. (2023). Effect of nutritional promotion intervention on dietary adherence among type II diabetes patients in North Shoa Zone Amhara Region: quasi-experimental study. J Health Popul Nutr.

[11] Lamptey R., Amoakoh-Coleman M., Barker M.M. (2023). Change in glycaemic control with structured diabetes self-management education in urban low-resource settings: multicentre randomised trial of effectiveness. BMC Health Serv Res.

[12] Muchiri J.W., Gericke G.J., Rheeder P. (2016). Impact of nutrition education on diabetes knowledge and attitudes of adults with type 2 diabetes living in a resource-limited setting in South Africa: a randomised controlled trial. J Endocrinol Metab Diabetes S Afr.

[13] Githinji P., Dawson J.A., Appiah D., Rethorst CD. (2022). A culturally sensitive and theory-based intervention on prevention and management of diabetes: a cluster randomized control trial. Nutrients.

[14] Gathu C.W., Shabani J., Kunyiha N., Ratansi R. (2018). Effect of diabetes self-management education on glycaemic control among type 2 diabetic patients at a family medicine clinic in Kenya: a randomised controlled trial. Afr J Prim Health Care Fam Med.

[15] Diriba D.C., Leung D.Y.P., Suen LKP. (2021). The effects of diabetes self-management interventions on physiological outcomes in people living with diabetes in Africa: a systematic review and meta-analysis. Diabet Med.

[16] Essien O., Otu A., Umoh V., Enang O., Hicks J.P., Walley J. (2017). Intensive patient education improves glycaemic control in diabetes compared to conventional education: a randomised controlled trial in a Nigerian Tertiary Care Hospital. PLOS ONE.

[17] Muchiri J.W., Gericke G.J., Rheeder P. (2021). Effectiveness of an adapted diabetes nutrition education program on clinical status, dietary behaviors, and behavior mediators in adults with type 2 diabetes: a randomized controlled trial. J Diabetes Metab Disord.

[18] Ojewale L.Y., Oluwatosin O.A. (2022). Family-integrated diabetes education for individuals with diabetes in South-west Nigeria. Ghana Med J.

[19] Ng’ang’a L., Ngoga G., Dusabeyezu S. (2022). Feasibility and effectiveness of self-monitoring of blood glucose among insulin-dependent patients with type 2 diabetes: open randomized control trial in three rural districts in Rwanda. BMC Endocr Disord.

[20] Abaza H., Marschollek M. (2017). SMS education for the promotion of diabetes self-management in low & middle income countries: a pilot randomized controlled trial in Egypt. BMC Public Health.

[21] Kiarie J.N., Mambo S.N., Kamundi G.K. (2024). A Quasi-experimental study on the effectiveness of primary caregiver social support capacity on self-management practices of clients living with type II diabetes (T2D) in Machakos County, Kenya. Int J Community Med Public Health.

[22] David E.A., Soremekun R.O., Abah I.O., Aderemi-Williams R.I. (2021). Impact of pharmacist-led care on glycaemic control of patients with uncontrolled type 2 diabetes: a randomised controlled trial in Nigeria. Pharm Pract.

[23] Hailu F.B., Moen A., Hjortdahl P. (2019). Diabetes self-management education (DSME) – effect on knowledge, self-care behavior, and self-efficacy among type 2 diabetes patients in Ethiopia: a controlled clinical trial. Diabetes Metab Syndr Obes Targets Ther.

[24] Hailu F.B., Hjortdahl P., Moen A. (2021). Effect of locally-contextualized nurse-led diabetes self-management education on psychosocial health and quality of life: A controlled before-and-after study. Int J Afr Nurs Sci.

[25] Tamiru S., Dugassa M., Amsalu B, Bidira K., Bacha L., Tsegaye D. (2023). Effects of Nurse-Led diabetes Self-Management education on Self-Care knowledge and Self-Care behavior among adult patients with type 2 diabetes mellitus attending diabetes follow-up clinic: a Quasi-Experimental study design. Int J Afr Nurs Sci.

[26] Anderson R.M., Funnell MM. (2008). The art and science of diabetes education: a culture out of balance. Diabetes Educ.

[27] Chowdhury H.A., Harrison C.L., Siddiquea B.N. (2024). The effectiveness of diabetes self-management education intervention on glycaemic control and cardiometabolic risk in adults with type 2 diabetes in low- and middle-income countries: a systematic review and meta-analysis. PLoS One.

[28] Alliston P., Jovkovic M., Khalid S., Fitzpatrick-Lewis D., Ali M.U., Sherifali D. (2024). The effects of diabetes self-management programs on clinical and patient reported outcomes in older adults: a systematic review and meta-analysis. Front Clin Diabetes Healthc.

[29] Fitzpatrick R., Pant S., Li J. (2023). Implementation of non-insulin-dependent diabetes self-management education (DSME) in LMICs: a systematic review of cost, adoption, acceptability, and fidelity in resource-constrained settings. Front Health Serv.

[30] Free C., Phillips G., Watson L. (2013). The effectiveness of mobile-health technologies to improve health care service delivery processes: a systematic review and meta-analysis. PLoS Med.

