# Supplementary material for: Effectiveness of Diabetes Self-Management Education and Support on Glycemic Control and Diabetes-Related Outcomes in Africa: A Systematic Review and Meta-Analysis
Source: AJPM Focus. 2025 Aug 20;5(4):100416. doi: 10.1016/j.focus.2025.100416 (PMC13377443; doi:10.1016/j.focus.2025.100416)
Supplement: Supplementary file 1 [file mmc1.docx]

**Appendix Table 1:** List of excluded studies and reasons

| **S/N** | **Author/Year** | **Study Title** | **Reason for Exclusion** |
| --- | --- | --- | --- |
| 1. | Wu 2023 | Effects of a Motivational Interview  Intervention on Self-Efficacy, Self-Care Behavior, and Glycemic Control in  Type 2 Diabetic Patients | Wrong setting |
| 2. | Flood 2020 | Health system interventions for adults with type 2 diabetes in low- and  middle-income countries: A systematic review and meta-analysis | Wrong study design |
| 3. | Derakhshandeh- Rishehri 2021 | The effects of formal nutrition  education on anthropometric indices, lipid profile, and glycemic control of patients with type 2 diabetes: a  systematic review and meta-analysis | Wrong study design |
| 4. | Pienaar 2020 | Self-management in face-to-face peer support for adults with type 2 diabetes living in low- or middle-income  countries: a systematic review | Wrong study design |
| 5. | Kumah 2021 | Influence of the Model of Care on the Outcomes of Diabetes Self-  Management Education Program: A Scoping Review | Wrong study design |
| 6. | Evans 2023 | Evaluating the effectiveness of  community health worker interventions on glycaemic control in type 2 diabetes: a systematic review and meta-analysis | Wrong study design |
| 7. | Hisham 2020 | Exercise as Component of Standard  Diabetes Management, the Challenges to Improve Performance among  Sudanese Type 2 Diabetes | Wrong study design |
| 8. | Bajaj 2018 | RSSDI clinical practice  recommendations for the management of type 2 diabetes mellitus 2017 | Wrong study design |
| 9. | Adam 2021 | Dietary knowledge, attitude and  practice among type 2 diabetes mellitus patients in Sudan: a hospital-based cross-sectional study | Wrong study design |
| 10. | Pathan 2023 | Diabetes care and education training  audit for primary care physicians across 47 counties of Kenya: A pre-post  intervention study | Wrong patient population |
| 11. | Lamptey 2022 | Cultural adaptation of a diabetes self- management education and support (DSMES) programme for two low  resource urban settings in Ghana, during the COVID-19 era | Wrong outcomes |
| 12. | Vukmirović 2023 | Perceptions of pharmacists and other healthcare professionals on marketed | wrong population |

|  |  | mobile applications used for self- management by type 2 diabetic  patients: A systematic review |  |
| --- | --- | --- | --- |
| 13. | Gucciardi 2019 | Emerging practices supporting diabetes self-management among food insecure adults and families: A scoping review | Wrong study design |
| 14. | Degefa 2020 | Predictors of Adherence Toward  Specific Domains of Diabetic Self-Care Among Type-2 Diabetes Patients | Wrong study design |
| 15. | Kobamo 2023 | Self-care Practice and associated  factors among Adults with Diabetes on Chronic Care Follow-up at Yirgalem  General Hospital, Sidama, Ethiopia: A cross-sectional study | Wrong study design |
| 16. | Hempler 2023 | Improving Health and Diabetes Self- Management in Immigrants with Type 2 Diabetes Through a Co-Created  Diabetes Self-Management Education and Support Intervention | Wrong setting |
| 17. | Kolb 2022 | An Effective Model of Diabetes Care and Education: The ADCES7 Self-Care Behaviors | Wrong study design |
| 18. | Nakidde 2022 | Knowledge and Practices of Self-Care among People with Diabetes in South Western Uganda: A Cross-Sectional Study at a Regional Referral Hospital in Mbarara City.: Diabetes self-care  knowledge and practice in Mbarara city. | Wrong study design |
| 19. | Nwankwo 2015 | Implementing diabetes self-  Management education (DSME) in a Nigerian population: perceptions of practice nurses and dieticians | Wrong Population |
| 20. | Bamuya 2021 | Use of the socio-ecological model to explore factors that influence the  implementation of a diabetes structured education programme (EXTEND  project) inLilongwe, Malawi and Maputo, Mozambique: a qualitative study | Wrong study design |
| 21. | Ojewale 2021 | Diabetes Self-Efficacy and Associated Factors among People Living with  Diabetes in Ibadan, Southwestern Nigeria | Wrong study design |
| 22. | Zwane 2023 | Self-Management of Diabetes and Associated Factors among Patients Seeking Chronic Care in Tshwane,  South Africa: A Facility-Based Study | Wrong study design |

| 23. | Anjali 2023 | Effectiveness of Diabetes Self-  Management Education on Distress and HbA1C among Indian Type 2 Diabetes Mellitus Patients: A Randomized  Controlled Trial | Wrong setting |
| --- | --- | --- | --- |
| 24. | Piotie 2021 | Designing an integrated, nurse-driven and home-based digital intervention to improve insulin management in under- resourced settings | Wrong  intervention |
| 25. | Ng'ang'a 2020 | Implementation of blood glucose self- monitoring among insulin-dependent patients with type 2 diabetes in three rural districts in Rwanda: 6 months  open randomised controlled trial | Wrong  intervention |
| 26. | Simegn 2023 | Adherence to Self - Care Practice Among Type 2 Diabetes Mellitus  Patients Using the Theory of Planned Behavior and Health Belief Model at Comprehensive Specialized Hospitals of Amhara Region, Ethiopia: Mixed  Method | Wrong study design |
| 27. | McElfish 2019 | Comparative Effectiveness and Maintenance of Diabetes Self-  Management Education Interventions for Marshallese Patients With Type 2 Diabetes: A Randomized Controlled Trial. | Wrong setting |
| 28. | Oraibi 2023 | Effectiveness of Blood Glucose Time in Range to Reduce Risk of Blood  Glucose Extrusion and Improve Blood Glucose Metrics in Type 1 Diabetic Patients | Wrong patient population |
| 29. | Kartika 2021 | Diabetic Self-Management Education – Effect on Self-Management Care of  Type-2 Diabetic Patients | Wrong setting |
| 30. | Yu 2022 | The effects of a nurse-led integrative medicine-based structured education  program on self-management behaviors among individuals with newly  diagnosed type 2 diabetes: a randomized controlled trial | Wrong setting |
| 31. | Oluchina 2022 | The effectiveness of an education  intervention based on self-care model on diabetes self-management behaviors and glycemic control | Wrong setting |
| 32. | Werfalli 2020 | Does social support effect knowledge and diabetes self-management practices in older persons with Type 2 diabetes  attending primary care clinics in Cape Town, South Africa? | Wrong study design |

| 33. | Kumah 2023 | Diabetes self-management education interventions in the WHO African Region: A scoping review | Wrong study design |
| --- | --- | --- | --- |
| 34. | Getrude Mphwanthe 2019 | Dietary and associated determinants of glycemic control and type 2 diabetes  self-management among adults in Malawi | Wrong study design |
| 35. | Lamptey 2022 | The effect of structured diabetes self-management education care on glycaemic control in Accra subsequent to COVID-19 | Wrong outcomes |
| 36. | Hailu 2018 | Nurse-Led Diabetes Self-Management Education Improves Clinical  Parameters in Ethiopia | Wrong intervention |
| 37. | Reyes 2023 | Glucometrics knowledge and its  relationship to glycemic control in  people living with type 1 diabetes: The GluKometrics study: Glucometrics  knowledge and glycemic control in type 1 diabetes | Wrong patient population |
| 38. | Lamptey 2022 | Diabetes self-management education interventions and self-management in  low-resource settings; a mixed-methods study | Wrong study design |

**Appendix Table 2:** Quality Assessment Result

| **Author (Year)** | **Sequence Generation** | **Allocation concealment** | **Blinding of participants and personnel for All outcomes** | **Blinding of outcome assessors for All outcomes** | **Incomplete outcome data for All outcomes** | **Selective outcome reporting** | **Other sources of bias for All outcomes** |  | | |
| --- | --- | --- | --- | --- | --- | --- | --- | --- | --- | --- |
| Lamptey (2023) | **+** | **?** | **+** | **+** | **+** | **+** | **+** |  |  |  |
| Gathu (2018) | **+** | **+** | **?** | **+** | **+** | **+** | **+** |  | **+** | Low risk |
| Muchiri (2016) | **+** | **+** | **?** | **+** | **+** | **+** | **+** |  | **?** | Unclear |
| Muchiri (2015) | **+** | **+** | **+** | **+** | **+** | **+** | **+** |  | **H** | High risk |
| Essien (2017) | **+** | **+** | **+** | **+** | **+** | **+** | **+** |  | | |
| Hailu (2019) | **+** | **+** | **H** | **H** | **+** | **+** | **+** |  |  |  |
| Muchiri (2021) | **+** | **+** | **+** | **+** | **+** | **+** | **+** |  |  |  |
| Hailu (2021) | **+** | **H** | **H** | **H** | **+** | **+** | **+** |  |  |  |
| David (2021) | **?** | **H** | **+** | **H** | **+** | **+** | **+** |  |  |  |
| Ojewale (2022) | **?** | **?** | **H** | **H** | **+** | **+** | **+** |  |  |  |
| Diriba (2023) | **+** | **+** | **+** | **H** | **+** | **+** | **+** |  |  |  |
| Eshete (2023) | **+** | **?** | **+** | **+** | **+** | **+** | **+** |  |  |  |
| Kiarie (2024) | **+** | **+** | **H** | **H** | **+** | **+** | **+** |  |  |  |
| Debussche (2018) | **+** | **+** | **+** | **+** | **+** | **+** | **+** |  |  |  |
| Ng'ang'a (2022) | **+** | **+** | **+** | **+** | **+** | **+** | **+** |  |  |  |
| Abaza (2017) | **+** | **+** | **+** | **+** | **+** | **+** | **+** |  |  |  |
| Githinji (2022) | **+** | **+** | **H** | **H** | **+** | **+** | **+** |  |  |  |
| Semegn (2023) | **+** | **H** | **H** | **H** | **+** | **+** | **+** |  |  |  |

**Appendix Figure 1**


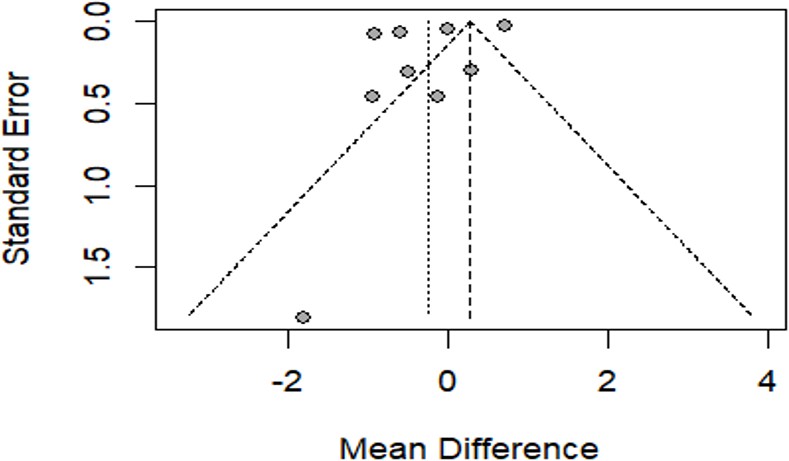


Supplementary Figure 1: Funnel Plot
